# Evaluation of endothelial function and subclinical atherosclerosis in association with hepatitis C virus in HIV-infected patients: a cross-sectional study

**DOI:** 10.1186/1471-2334-11-265

**Published:** 2011-10-03

**Authors:** Mar Masiá, Sergio Padilla, Catalina Robledano, José M Ramos, Félix Gutiérrez

**Affiliations:** 1 Infectious Diseases Unit, Hospital General Universitario de Elche, Alicante, Spain; 2 Department of Clinical Medicine, University Miguel Hernández, Elche, Alicante, Spain

## Abstract

**Background:**

Relationship of hepatitis C virus (HCV) infection with an increased risk of cardiovascular disease (CVD) in HIV-infected patients remains controversial. We evaluated endothelial function and subclinical atherosclerosis in HIV-infected patients with and without HCV.

**Methods:**

Flow-mediated dilatation (FMD) of the brachial artery and circulating levels of cell adhesion molecules (CAM) were measured in HCV/HIV-coinfected and HIV-monoinfected patients. Subclinical atherosclerosis was assessed by carotid intima-media thickness (cIMT).

**Results:**

63 (31%) HCV/HIV-coinfected and 138 (69%) HIV-monoinfected patients were included. Median soluble vascular CAM-1 (sVCAM-1) and intercellular CAM-1 (sICAM-1) levels were significantly higher in HIV/HCV-coinfected patients (P < 0.001 for both cases). Median (interquartile range) FMD was 6.21% (2.86-9.62) in HCV/HIV-coinfected and 5.54% (2.13-9.13) in HIV-monoinfected patients (P = 0.37). Adjustment for variables associated with HCV and FMD disclosed similar results. FMD correlated inversely with cIMT and age. Carotid IMT did not differ between HCV/HIV-coinfected and HIV-monoinfected patients in unadjusted (0.61 [0.55-0.65] mm vs 0.60 [0.53-0.72] mm; P = 0.39) or adjusted analyses.

**Conclusion:**

HCV infection was associated with higher levels of sICAM-1 and sVCAM-1, but no evidence of increased subclinical atherosclerosis was found when endothelial function was evaluated through FMD, or when assessing the cIMT.

## Background

Relationship of hepatitis C virus (HCV) infection with an increased risk of cardiovascular disease (CVD) in HIV-infected patients remains controversial. While data from large cohort studies support a higher frequency of cardiovascular events in these patients [1,2], other studies have shown differing results [3], and no increased subclinical atherosclerosis measured with the carotid intima-media thickness (cIMT) was found in a large cohort of HCV/HIV-coinfected compared with HIV-monoinfected women [4].

Endothelial dysfunction (ED) is an early event in the development of atherosclerosis [5,6]. HCV/HIV-coinfection has been associated with ED in a study based in the measurement of circulating cell adhesion molecules (CAM) levels [7]. Likewise, a sustained decrease in CAM levels (intercellular CAM-1 [ICAM-1] and vascular CAM-1 [VCAM-1]) has been described following therapy for HCV with pegylated interferon plus ribavirin [8]. At present, the non-invasive technique of choice to assess ED is flow-mediated dilatation (FMD) of the brachial artery [9,10], a validated test that has shown to be related to the prevalence and extent of coronary atherosclerosis [11], and to predict future cardiovascular events [12]. To date, no studies have measured brachial FMD to assess the risk of future CVD development in HCV-infected patients.

We aimed to evaluate ED through FMD of the brachial artery and subclinical atherosclerosis through cIMT in a cohort of HIV-infected patients with and without HCV coinfection.

## Methods

### Setting and inclusion/exclusion criteria

The investigation was conducted at the HIV Outpatient Clinic of the University General Hospital of Elche, Spain. All patients visited during a four-month period (February-June 2009) were invited to participate in this cross-sectional study. Eligible subjects included HIV-infected adults aged 18-75 years, whether they were coinfected with HCV or not, and with no changes in their antiretroviral regimen or cardiovascular risk factor therapy during the last 6 months. Exclusion criteria were active infections, negative HCV RNA or positive hepatitis B surface antigenemia in HCV-coinfected patients, HCV previous therapy with sustained response, and pregnancy. The study was approved by the Hospital General Universitario de Elche Ethics Committee (CEIC), and all the patients gave their informed consent.

### Clinical and laboratory measurements

Details were taken of age, HIV-related data, cardiovascular risk factors, lipodystrophy, and hepatitis B virus coinfection. HCV infection was defined by a positive HCV antibody assay and a confirmatory positive HCV RNA. HCV genotyping (sequencing) was performed. Dyslipidemia, diabetes and hypertension were defined by a previous diagnosis reported by the patient and/or recorded in the patients' charts, or by a current prescription of pharmacological therapy for any of such risk factors. Patients on antiretroviral therapy and/or cardiovascular risk factor therapy had to be on a stable treatment regimen for at least 6 months to be included. Lipodystrophy was defined as the presence of body-fat changes that could be clearly recognised by both the patient and the doctor. The liver fibrosis scores APRI and FIB-4 were calculated according to the proposed formulas (Table 1).

**Table 1:** Characteristics of the patients with and without hepatitis C coinfection

Variable	Hepatitis C virus + (N = 63)	Hepatitis C virus - (N = 138)	P
Age, years (median [IQR])	43.7 (39.4-47.0)	43.5 (35.6-49.5)	0.55
Men, no. (%)	53 (84.1)	100 (72.5)	0.08
Category C of the CDC, no. (%)	13 (20.6)	49 (35.5)	0.04
IDU, no. (%)	58 (92.1)	11 (8.0)	<0.001
MSM, no. (%)	0	56 (40.6)	<0.001
Viral load < 50 copies/mL, no. (%)	44 (69.8)	90 (65.2)	0.63
CD4 cell count, cells/μL (median [IQR])	530 (280-750)	495 (317.5-652.5)	0.86
On antiretroviral therapy, no. (%)	56 (88.9)	115 (83.3)	0.39
Duration of antiretroviral therapy exposure, years (median [IQR])	9.0 (3.75-10.0)	6.0 (1.88-10.0)	0.25
Non nucleoside analog-based regimen, no. (%)	17 (27.0)	53 (38.7)	0.11
Protease inhibitor-based regimen, no. (%)	36 (57.1)	58 (42.3)	0.07
Lipodystrophy^1^, no. (%)	19 (30.2)	47 (34.1)	0.63
Current smoker, no. (%)	53 (84.1)	71 (51.4)	<0.001
Dyslipidemia, no. (%)	12 (19)	56 (40.6)	0.002
Lipid-lowering therapy	5 (7.9)	42 (30.4)	<0.001
Hypertension, no. (%)	8 (12.7)	29 (21.0)	0.17
Diabetes, no. (%)	3 (4.8)	14 (10.1)	0.28
Pre-existing cardiovascular disease, no. (%)	2 (3.2)	3 (2.2)	0.65
Body mass index, Kg/m2 (median [IQR])	24.05 (21.56-27.86)	25.0 (21.99-28.32)	0.48
Hepatitis B virus, no. (%)	0	4 (3.0)	0.30
Hepatitis C viral load, copies/mL (median [IQR])	748,056 (145,564-3,204,245)	-	-
APRI index (median [IQR])	0.67 (0.39-1.29)	0.22 (0.18-0.31)	<0.001
Genotypes 1 and 4 (n = 37)	0.67 (0.40-1.11)		
Genotypes 2 and 3 (n = 24)	0.73 (0.38-1.62)		0.93^3^
FIB-4 index (median [IQR])	1.40 (0.98-3.27)	0.91 (0.66-1.17)	<0.001
Genotypes 1 and 4 (n = 37)	1.52 (1.09-2.74)		
Genotypes 2 and 3 (n = 24)	1.42 (0.91-4.14)		0.86^3^
Waist-to-hip ratio (median [IQR])	0.94 (0.89-0.98)	0.93 (0.88-0.98)	0.72
Metabolic syndrome^2^, no. (%)	14 (22.2)	42 (30.4)	0.31
Framingham risk at 10 years^4^, % (median [IQR])	5 (2-8)	4 (1-10)	0.69
Systolic blood pressure, mm Hg (median [IQR])	122 (114-133)	130 (118.5-142)	0.006
Diastolic blood pressure, mm Hg (median [IQR])	76 (69-83)	79 (71-85)	0.21
Fasting total cholesterol, mg/dL (median [IQR])	163 (146-186)	185 (158.8-213.3)	0.001
Fasting LDL-cholesterol, mg/dL (median [IQR])	104 (86-123)	119.5 (100-140)	0.001
Fasting HDL-cholesterol, mg/dL (median [IQR])	41.2 (34.5-50.2)	43.5 (36.9-51.2)	0.56
Fasting triglycerides, mg/dL (median [IQR])	131 (86-171)	137.5 (94.8-197.5)	0.30
Fasting glucose, mg/dL (median [IQR])	87.0 (81-99)	87.0 (80.8-97)	0.93
VCAM-1, ηg/mL (median [IQR])	931.4 (707.8-1606.5)	766.6 (630.3-974.5)	<0.001
ICAM-1, ηg/mL (median [IQR])	492.6 (354.4-713.1)	314.3 (251.9-408.3)	<0.001
Baseline brachial artery diameter, cm (median [IQR])	4.30 (3.87-4.53)	4.14 (3.60-4.60)	0.20
Brachial artery FMD, % (median [IQR])	6.21 (2.86-9.62)	5.54 (2.13-9.13)	0.37
Genotypes 1 and 4 (n = 37)	6.78 (2.01-11.80)		
Genotypes 2 and 3 (n = 24)	5.09 (3.02-8.29)		0.32^3^
Brachial artery nitroglycerine MD, % (median [IQR])	18.35 (15.27-25.65)	19.68 (12.31-24.57)	0.86
Genotypes 1 and 4 (n = 37)	19.44 (15.50-28.16)		
Genotypes 2 and 3 (n = 24)	18.22 (11.62-22.08)		0.16^3^
Carotid IMT, mm (median [IQR])	0.61 (0.55-0.65)	0.60 (0.53-0.72)	0.39
Genotypes 1 and 4 (n = 37)	0.59 (0.52-0.65)		
Genotypes 2 and 3 (n = 24)	0.63 (0.55-0.68)		0.15^3^
Carotid plaques, no. (%)	25 (39.7)	48 (38.4)	0.53

All continuous variables are expressed as median (interquartile range). Categorical variables represent number (%).

IDU, intravenous drug users; MSM, men who have sex with men; FMD, flow-mediated dilatation; MD, mediated dilatation; IMT, intima-media thickness; IQR, interquartile range.

APRI = [AST (U/L)/upper limit of normal (U/L)] × 100/platelets (10^9^/L).

FIB-4 = Age × AST (U/L)/[platelets (10^9^/L) × ALT^1/2 ^(U/L)]

^1^Lipodystrophy was defined as the presence of body-fat changes that could be clearly recognised by both the patient and the doctor.

^2^Metabolic syndrome was defined according to the new International Diabetes Federation criteria. by the existence of central obesity (waist circumference in females > 80 cm and in males > 94 cm in Europeans) plus two of the following factors: raised triglycerides (> 150 mg/dl), reduced HDL cholesterol (< 50 mg/dl in females and < 40 mg/dl in males), raised blood pressure (≥130/85 mm Hg), and raised fasting plasma glucose (> 100 mg/dl), or specific therapy for any of such conditions.

^3^P for the comparison between genotypes 1 and 4 with genotypes 2 and 3.

^4^The 10-year risk of developing myocardial infarction or coronary death was calculated for each patient with the Framingham equation.

Blood samples were collected after an 8 hour overnight fast for measurement of glycaemia, total cholesterol, HDL-cholesterol, direct LDL-cholesterol, triglycerides, creatinine, CD4 cell count and HIV plasma viral load. An additional sample was processed by centrifugation. Plasma aliquots obtained were stored at -80°C. All frozen samples were subsequently defrosted, and plasma levels of soluble vascular cell adhesion molecule-1 (sVCAM-1) and soluble intercellular adhesion molecule-1 (sICAM-1) were measured using commercially available ELISA kits (Quantikine, R&D Systems Europe Ltd, UK).

### Evaluation of endothelial function by flow-mediated dilatation of the brachial artery

Endothelial function was evaluated by measuring FMD with a high resolution linear array vascular ultrasound transducer (Nemio XG, Toshiba Medical Systems SA) as previously described [13]. Subjects were required to be fasting and not use any tobacco-containing products for 8 hours before the study. Baseline vessel diameter was determined with the mean of three measures. Then, a forearm cuff was inflated to 240 mm Hg for 5 minutes. Diameter of the brachial artery was measured 1 min after cuff deflation. FMD was calculated as the ratio of the brachial artery diameter after reactive hyperemia to baseline diameter and was expressed as a percentage of change. Nitroglycerin-mediated vasodilatation was also determined. Measurements were performed by a single sonographist blinded to the subject's information. Reproducibility was assessed in 10 healthy subjects (8 men, age 44 ± 8,5 years) who were examined by the same sonographer twice, at an interval of 2 hours. The median (interquartile range [IQR]) intraobserver intersession percentage of variation for brachial artery diameter was 2.38% (0-5.71), similar to previous studies [14].

### Evaluation of the carotid intima-media thickness

For determination of the carotid IMT, B-mode high resolution ultrasound was used following a standard procedure as described previously [15]. To quantify the degree of carotid artery wall thickening, the mean of six measures performed in the posterior wall of the left and right common carotid arteries was taken. Carotid plaques were defined as a focal cIMT≥1 mm in any of the imaged segments.

### Statistical analysis

Differences in demographic and clinical characteristics between patients with and without HCV infection were assessed using the chi-squared or Fisher's exact test for categorical variables and Mann-Whitney test for continuous variables. Multivariable linear regression analyses were performed to quantify the association of HCV infection with the logarithm of FMD, CAM and cIMT after adjustment for potential confounding factors. Potential confounders included all variables significantly associated with hepatitis C virus infection in univariate analysis, or with a trend to statistical significance (P < 0.10): sex, C CDC category, intravenous drug use, men who have sex with men, current protease inhibitor use, lipid-lowering therapy, smoking habit, systolic blood pressure, fasting total cholesterol and LDL-cholesterol, APRI and FIB-4. Additionally, to assess the effect of HCV on FMD values, all variables associated with FMD in univariate analysis, as well as baseline diameter of brachial artery, were also included as potential confounders in this case: age, hypertension, diabetes, and lipodystrophy. The same procedure was used to quantify the effect of the genotypes 1 and 4 versus 2 and 3 on FMD, CAM and cIMT. All P values were two-tailed. It was calculated that 70 HCV/HIV coinfected patients and 140 HIV-monoinfected would be required to detect an absolute FMD difference between groups of 2% (standard deviation 5%) with an 86% statistical power, alpha 0.05. The final statistical power of this study including 63 and 138 patients in each group was 83.8%.

## Results

Two hundred and fourteen patients were initially included. Of 76 patients with a positive HCV antibody assay, 5 patients were excluded because of negative HCV RNA, 5 patients because of previous therapy for HCV and a negative HCV viral load, and 3 patients due to HCV infection with positive HBV antigenemia, leaving 63 (31%) patients with HCV/HIV coinfection and 138 (69%) HIV-monoinfected patients. Characteristics of the patients are listed in Table 1. Patients with HCV/HIV coinfection were more frequently intravenous drug users, were less likely to belong to the C CDC category, and tended to receive more frequently protease inhibitors and to be men. Among traditional cardiovascular risk factors, patients with HCV/HIV coinfection were more frequently smokers, and had a lower prevalence of dyslipidemia, lipid-lowering therapy, lower systolic blood pressure, lower total cholesterol, and LDL-cholesterol.

Median sVCAM-1 and sICAM-1 levels were significantly higher in HIV/HCV-coinfected than in HIV-monoinfected patients (P < 0.001 for both cases, Table 1). When adjustment was performed for the variables associated with HCV (see Table 2), sICAM-1 levels were still 62.4% higher in HIV/HCV coinfected patients (P < 0.001), and sVCAM-1 tended to be higher (33.1%, P = 0.08) (Table 2). sICAM-1 continued to be higher in HCV-coinfected individuals when only those with an HIV viral load < 50 copies/ml were selected (Table 2). There was a high correlation of sICAM-1 and sVCAM-1 with APRI (Spearman's rho = 0.42 and 0.45 respectively) and FIB-4 Spearman's rho = 0.34 and 0.39 respectively) fibrosis indexes (P < 0.001 in all cases). HCV viral load correlated with sVCAM-1 (Spearman's rho 0.36, P = 0.008), and to a lesser extent with sICAM-1 (Spearman's rho 0.25, P = 0.07). No differences between genotypes were found for sVCAM-1 or sICAM-1 in unadjusted analyses, nor after adjustment for variables associated with HCV (P = 0.66 and P = 0.46 respectively).

**Table 2:** Association of HIV and HIV/HCV infection with flow-mediated dilatation, carotid intima-media thickness, and circulating levels of cell adhesion molecules

Variable	Mean difference in log brachial artery FMD, % (95% CI)	P	Mean difference in log carotid IMT, mm (95% CI)	P	Mean difference in log sICAM-1, ηg/mL (95% CI)	P	Mean difference in log sVCAM-1, ηg/mL (95% CI)	P
**HIV/HCV vs. HIV**								
Unadjusted	0.10 (-0.18-0.38)	0.47	-0.05 (-0.11-0.017)	0.15	0.36 (0.22-0.50)	< 0.001	0.23 (-0.10-0.55)	0.01
Adjusted	0.17 (-0.33-0.68)	0.50^1^	0.05 (-0.05-0.16)	0.27^3^	0.39 (0.14-0.64)	0.002^3^	0.22 (0.05-0.39)	0.17^3^
**HIV/HCV vs. HIV** **(patients with VL < 50 c/mL)**^2^								
Unadjusted	0.009 (-0.33-0.34)	0.96	-0.08 (-0.16-0.003)	0.06	0.33 (0.14-0.52)	0.001	0.17 (-0.06-0.40)	0.14
Adjusted	0.23 (-0.45-0.90)	0.47^1^	0.014 (-0.13-0.16)	0.24^3^	0.55 (0.19-0.90)	0.003^3^	0.21 (-0.28-0.69)	0.25^3^

^1^Adjusted for baseline brachial artery diameter, age, sex, C CDC category, intravenous drug use, men who have sex with men, current protease inhibitor use, lipid-lowering therapy, hypertension, diabetes, smoking, lipodystrophy, systolic blood pressure, fasting total cholesterol and LDL-cholesterol, APRI and FIB-4.

^2^N = 134

^3^Adjusted for sex, C CDC category, intravenous drug use, men who have sex with men, current protease inhibitor use, lipid-lowering therapy, smoking, systolic blood pressure, fasting total cholesterol and LDL-cholesterol, APRI and FIB-4.

FMD, flow-mediated dilatation; IMT, intima-media thickness; HCV, hepatitis C virus; CI, confidence interval

Median (IQR) FMD was 6.21% (2.86-9.62)% in patients HCV/HIV-coinfected and 5.54% (2.13-9.13)% in HIV-monoinfected patients (P = 0.37). No correlation was found between FMD and APRI or FIB-4 indexes. Factors associated with a lower FMD in univariate analysis were male sex (P = 0.001), dyslipidemia (P = 0.003), hypertension (P = 0.02), diabetes (P = 0.02), and lipodystrophy (P = 0.009). FMD correlated inversely with the cIMT (Spearman's rho = -0.32; P < 0.001) and with age (Spearman's rho = -0.23; P = 0.001).

Table 2 shows the effect of HCV coinfection on FMD values. After adjustment for the variables associated with HCV and with FMD (Table 2) and for baseline artery diameter, FMD remained not statistically different when patients HCV/HIV-coinfected and HIV-monoinfected were compared (17.23% higher FMD values in HCV/HIV-coinfected patients, P = 0.53). To avoid the effect of HIV viremia on the endothelial function, the same analyses were performed only in patients with undetectable viral load (< 50 copies/ml) (Table 2). FMD was again not different according to HCV status.

The influence of HCV genotype on FMD was also explored. FMD values did not differ between patients with genotypes 1/4 and 2/3 (P = 0.32). Adjusted analysis generated similar results (P = 0.49).

Carotid IMT did not differ between HCV/HIV-coinfected and HIV-monoinfected patients (0.61 [0.55-0.65] mm vs 0.60 [0.53-0.72] mm; P = 0.39). After adjustment for the variables associated with HCV, the difference between the two groups remained non-significant (Table 2). Analysis by genotypes yielded similar results, with no significant changes after adjustment (data not shown).

## Discussion

We found that there was little difference in brachial FMD between HCV/HIV-coinfected and HIV-monoinfected patients, despite higher circulating levels of CAM among HCV-coinfected individuals. Another surrogate marker of atherosclerosis such as the cIMT was also not different between groups. Patients with HCV/HIV coinfection from our cohort differed from the HIV-monoinfected in the prevalence of cardiovascular risk factors. HCV-coinfected individuals had less frequently dyslipidemia, had lower total and LDL-cholesterol levels, lower systolic blood pressure, and were less likely to belong to C CDC category, while they were more frequently smokers, and tended to be more often men, and to receive protease inhibitors. When all those protective and detrimental factors on cardiovascular risk were adjusted for, again not significant differences were found in endothelial function measured through brachial FMD or in the cIMT/plaque frequency between HCV/HIV-coinfected and HIV-monoinfected patients. Neither were there differences in FMD according to HCV genotype.

Our results show a pro-atherosclerotic profile in the vascular inflammation biomarkers sICAM-1 and sVCAM-1. These molecules have also been linked to hepatocellular injury, specifically sICAM-1 has been related to liver inflammatory activity and hepatocellular necrosis, and sVCAM-1 to liver fibrosis [16]. Accordingly, in our patients sICAM-1 and sVCAM-1 correlated closely with the liver fibrosis indexes APRI and FIB-4. In contrast, we did not find increased subclinical atherosclerosis when ED and cIMT were assessed in HCV-coinfected patients compared to HIV-monoinfected. These results are in agreement with those previously described with the cIMT in a cohort of HCV/HIV-coinfected women in the Women's Interagency HIV Study [4], and suggest a complex interaction among factors accounting for the risk of CVD development in HCV-coinfected patients. Whether additional aspects associated with HCV infection, such as a more favourable lipid profile [17] or further protective factors related to HCV or to IDU may counteract for the pro-atherogenic ones or if, on the contrary, other issues such as higher plaque instability linked with inflammation [18,19] or the pro-coagulant state associated with HCV [20,21] may contribute to a greater incidence of ischemic events in the absence of an overt higher atherosclerotic burden, merits further research.

Although currently the test of choice for ED evaluation, FMD measurement has inherent physiological variations, and this has to be taken into account when interpreting our results. Another limitation of the study is the cross-sectional design and the relatively small number of patients included; however, the sample size was in line with other studies evaluating FMD, a technically complex test, and indeed the study was powered enough to show that levels of ICAM-1 and VCAM-1 were significantly higher in HCV/HIV-coinfected patients. Additionally, this is the first study evaluating ED through FMD of the brachial artery in HCV/HIV-coinfected patients, with a representative sample of the HIV population cared for in an outpatient clinic. The inverse correlation between IMT and FMD supports the consistency of the measurements.

## Conclusion

In HIV-infected patients, HCV infection was associated with higher levels of ICAM-1 and VCAM-1, but no evidence of increased subclinical atherosclerosis was found when endothelial function was evaluated through brachial FMD, or when assessing the cIMT. Future prospective studies are warranted to clarify the influence of HCV infection on atherosclerotic disease in HIV-infected patients in order to optimize the management of these patients.

## Competing interests

The authors declare that they have no competing interests.

## Authors' contributions

FG and MM conceptualized and designed the study. CR acquired data and performed the laboratory analyses. MM, FG, SP and JR analyzed and interpreted the data and results. MM and FG drafted the manuscript. All authors discussed the results and commented on the manuscript and all read and approved the final manuscript

## Pre-publication history

The pre-publication history for this paper can be accessed here:

http://www.biomedcentral.com/1471-2334/11/265/prepub
